# Crystal structures of three *N*,*N*,*N*′-tris­ubstituted thio­ureas for reactivity-controlled nanocrystal synthesis

**DOI:** 10.1107/S2056989022000147

**Published:** 2022-01-14

**Authors:** Evert Dhaene, Isabel Van Driessche, Klaartje De Buysser, Kristof Van Hecke

**Affiliations:** a SCRiPTS group, Sol-gel Centre for Research on Inorganic Powders and Thin films Synthesis, Department of Chemistry, Ghent University, Krijgslaan 281-S3, B-9000 Ghent, Belgium; b XStruct, Department of Chemistry, Ghent University, Krijgslaan 281-S3, B-9000 Ghent, Belgium

**Keywords:** crystal structure, nanocrystals, thio­ureas

## Abstract

The synthesis and single-crystal X-ray structures of three *N*,*N*,*N*′-tris­ubstituted thio­ureas are reported, namely *N*,*N*,*N*′-tri­benzyl­thio­urea, C_22_H_22_N_2_S (**1**), *N*-methyl-*N*,*N*′-di­phenyl­thio­urea, C_14_H_14_N_2_S (**2**), and *N*,*N*-di-*n*-butyl-*N*′-phenylthio­urea, C_15_H_24_N_2_S (**3**). The influence of the different substituents on the thio­ureas is clear from the delocalization of the thio­urea C—N and C=S bonds, while the crystal structures show infinite chains of *N*,*N*,*N*′-tri­benzyl­thio­urea (**1**), hydrogen-bonded pairs of *N*-methyl-*N*,*N*′-di­phenyl­thio­urea (**2**) and hexa­mer ring assemblies of *N*,*N*-di-*n*-butyl-*N*′-phenylthio­urea (**3**) mol­ecules. The above-mentioned compounds were synthesized *via* a mild, general procedure, readily accessible precursors and with a high yield, providing straightforward access to a whole library of thio­ureas.

## Abstract 2

Crystal structures of three *N*,*N*,*N*′-tris­ubstituted thio­ureas, with varying substitution patterns, for reactivity-controlled nanocrystal synthesis are reported.

## Chemical context

To control the size of colloidal nanocrystals, many traditional methods terminate the synthesis during the nanocrystal growth at the desired size. However, this leads to a lower yield, higher size dispersity, and it is difficult to get a good reproducibility (Owen *et al.*, 2010 ▸; Abe *et al.*, 2012 ▸, 2013 ▸). Therefore, Owen *et al.* suggest a new method that uses a library of substituted thio­ureas, whose substitution pattern tunes their conversion reactivity (Hendricks *et al.*, 2015 ▸; Hamachi *et al.*, 2017 ▸). By this, the nanocrystal concentration can be adjusted and the desired nanocrystal size can be obtained at full conversion, with a high degree of consistency. This control is obtained by varying the substitution pattern of the thio­urea, and thus the conversion reactivity (Hens, 2015 ▸). This can be understood from the fact that the conversion reactivity is influenced by the number of substituents, and their electronic and steric properties. The conversion rate, *i.e.* reactivity, decreases as the number of substituents increases, or by replacing electron-withdrawing with electron-donating groups (*e.g.* substituting aryl for alkyl substituents). These thio­ureas are synthesized *via* a one-step click reaction between iso­thio­cyanates and primary or secondary amines (Hendricks *et al.*, 2015 ▸). In addition, they have a long shelf-life and are air-stable after synthesis (Hendricks *et al.*, 2015 ▸). An additional advantage of these precursors is that the starting reagents are relatively cheap and widely commercially available, in large qu­anti­ties. When added to a hot solution of metal oleate, such as lead, cadmium, zinc, *etc*., this results in the formation of highly reproducible, monodisperse, homogeneously capped metal sulfide nanocrystals at a full yield (Hendricks *et al.*, 2015 ▸; Hamachi *et al.*, 2017 ▸; Dhaene *et al.*, 2019 ▸). 
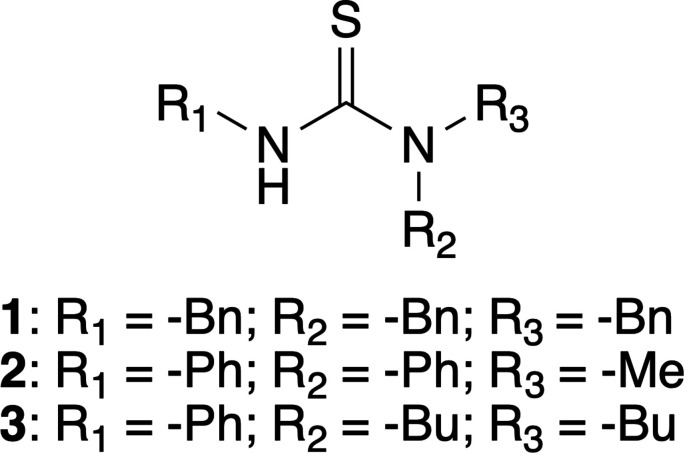


Herein, we report the single-crystal X-ray structural analysis of the following tris­ubstituted thio­ureas: *N*,*N*,*N*′-tri­benzyl­thio­urea (**1**), *N*-methyl-*N*,*N*′-di­phenyl­thio­urea (**2**), and *N*-phenyl-*N*′,*N*′-di-*n*-butyl­thio­urea (**3**), prepared *via* a simple, straightforward synthesis method making use of readily commercially available compounds, to a high purity (> 99%) and with a high yield (> 75%).

## Structural commentary

**Figure 1:**
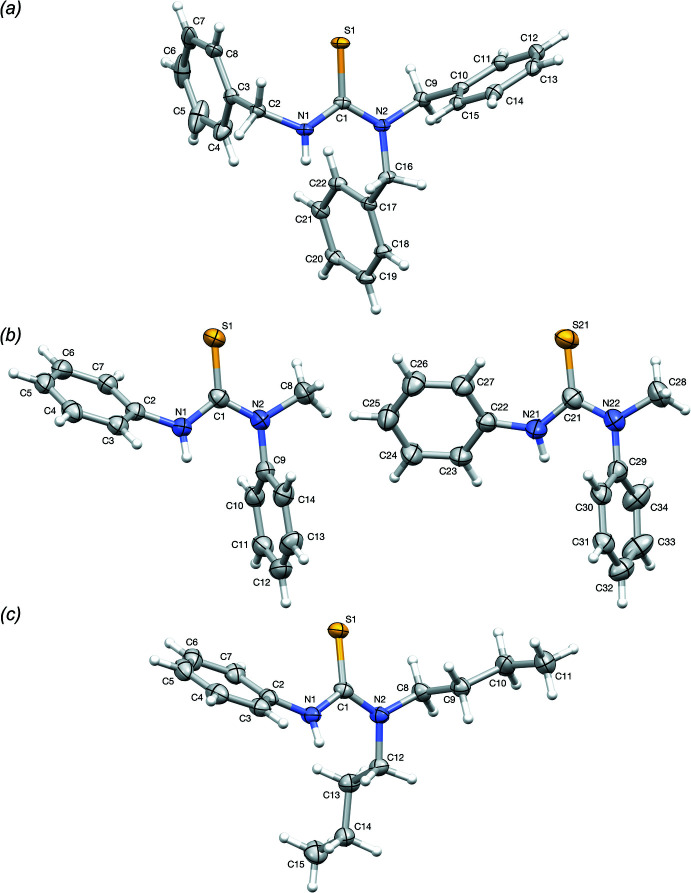
Mol­ecular structures of (*a*) **1**, (*b*) **2** and (*c*) **3**, showing thermal displacement ellipsoids drawn at the 50% probability level and the atom-labelling scheme for the non-hydrogen atoms. For **2**, both mol­ecules of the asymmetric unit are shown.

Compound **1** crystallizes in the centrosymmetric monoclinic space group *P*2_1_/c, with the asymmetric unit consisting of one *N*,*N*,*N*′-tri­benzyl­thio­urea mol­ecule. On the one hand, the secondary amine benzyl ring (C3–C8) is found to be almost completely parallel to one of the tertiary amine benzyl rings (C17–C22), subtending a dihedral angle of 8.92 (8)° between the best planes through the two benzene rings. On the other hand, the two tertiary amine benzyl rings (C10–C15 and C17–C22) are highly twisted to each other, with a dihedral angle of 76.96 (7)° between the best planes through the two benzene rings (Fig. 1 ▸ *a*). The N1—C1 and C1—N2 bond distances are 1.3419 (18) and 1.3569 (18) Å, respectively, while the C1=S1 (double) bond distance is 1.6905 (14) Å.

Compound **2** crystallizes in the centrosymmetric triclinic space group *P* 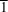 , with two *N*-methyl-*N*,*N*′-di­phenyl­thio­urea mol­ecules in the asymmetric unit. The secondary and tertiary amine phenyl rings (C2–C7, C9–C14 and C22–C27, C29–C34, for the first and second mol­ecules, respectively) subtend a dihedral angle of 69.39 (9) and 75.70 (10)°, respectively, between the best planes through the two phenyl rings (Fig. 1 ▸ *b*). The N1—C1 and C1—N2 bond distances are 1.359 (2) and 1.352 (3) Å, for mol­ecule 1, while the respective N21—C21 and C21—N22 bond distances are 1.367 (2) and 1.345 (3) Å, for mol­ecule 2. The C1=S1 and C21=S22 (double) bond distances are 1.6835 (17) and 1.6798 (19) Å, for mol­ecule 1 and 2, respectively.

**Figure 2:**
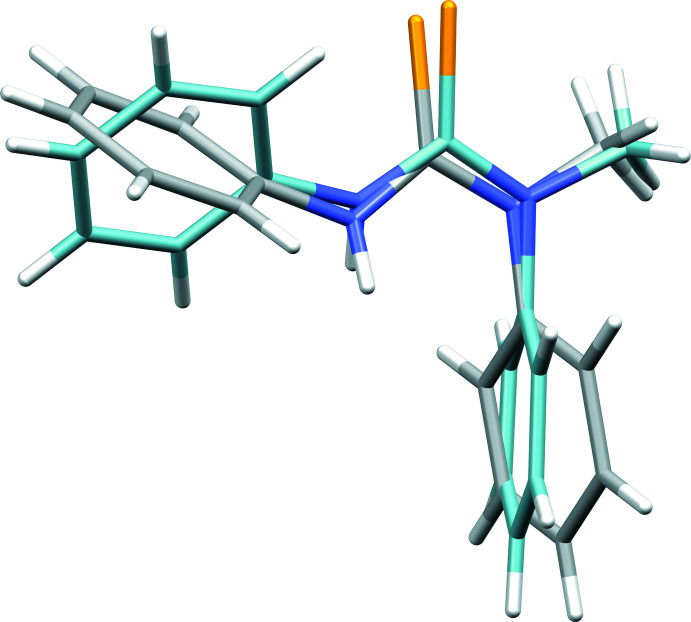
Fit of the first (grey) and second (light blue) mol­ecule in the asymmetric unit of **2**, showing an r.m.s.d. of 1.174 Å.

The influence of the two phenyl substituents on the delocalization of the N1—C1, C1—N2 and C1=S1 bonds is clear, in comparison with the structure of **1**, *i.e.* the lone electron pair on N1/N21 is more delocalized towards the secondary amine phenyl ring substituent in **2**, leading to an increased N1—C1/N21—C22 distance of 1.359 (2)/1.367 (2) Å, which is even more pronounced for the second mol­ecule in the asymmetric unit, because of higher planarity of the phenyl ring with the N—C(=S)—N plane (Fig. 2 ▸). However, the delocalization of N2/N22 is less pronounced towards the tertiary amine phenyl ring, with a C1—N2/C21—N22 distance of 1.352 (3)/1.345 (3) Å, because of the latter phenyl ring being almost perpendicular to the central N—C(=S)—N plane. As a consequence of the improved delocalization of N1/N21 in **2**, the C1=S1/C21=S21 bond length decreases slightly – although less significantly in the case of C1=S1 – to 1.6835 (17)/1.6798 (19) Å in comparison with **1**.

The structure of **3** has very recently been deposited with the Cambridge Structural Database (CSD) (refcode OYOSIH; Rahman *et al.*, 2021 ▸); however, the mentioned structure was determined at room temperature and showed disorder of both butyl substituents, as well as the presence of unknown solvent, which was treated by the SQUEEZE procedure in *PLATON* (Spek, 2015 ▸). Here, our reported structure was determined at 100 K and shows no signs of any kind of (solvent) disorder. The unknown solvate structure of Rahman *et al.* (2021 ▸) might be caused by the use of acetone as solvent and recrystallization by slow evaporation from EtOH, whereas we used toluene as solvent and recrystallized from a hot hexa­ne:EtOH (10:1) mixture by slowly cooling down. Compound **3** crystallizes in the trigonal space group *R* 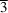 , with one *N*-phenyl-*N*′,*N*′-di-*n*-butyl­thio­urea mol­ecule in the asymmetric unit. The phenyl substituent on the secondary amine is twisted with respect to the central N—C—S—N plane, with a C1—N1—C2—C7 torsion angle of 55.54 (16)°, while the two butyl substituents are found completely staggered (Fig. 1 ▸ *c*). The N1—C1 and C1—N2 bond distances are 1.3594 (15) and 1.3432 (15) Å, respectively, while the C1=S2 (double) bond distance is 1.7004 (11) Å. The delocalization of N1 towards the secondary amine phenyl substituent is also noticed here, comparable to **2**, while there is minimal delocalization of N2 towards the butyl substituents, consequently showing the shortest C1—N2 and the longest C1=S1 distances.

## Supra­molecular features

**Figure 3:**
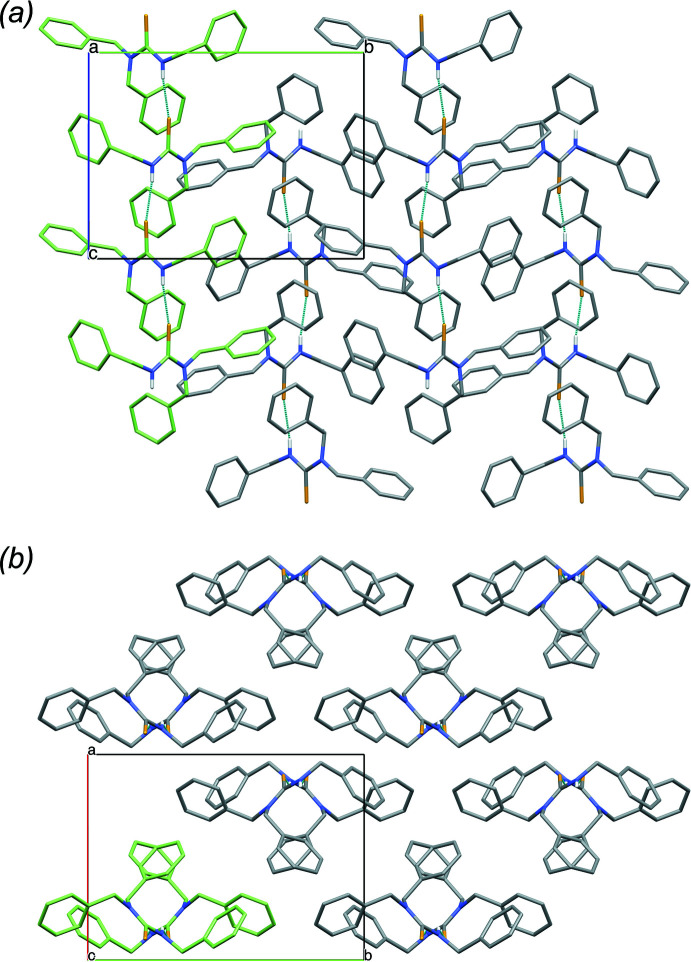
Packing in the structure of **1**, (*a*) viewed down the *a* axis, showing the N1—H1⋯S1 hydrogen bonds, linking the *N*,*N*,*N*′-tri­benzyl­thio­urea mol­ecules into infinite chains along the [001] direction, and (*b*) viewed down the *c* axis, showing the columnar arrangement through alternating orientations of the mol­ecules. A chain of four hydrogen-bonded mol­ecules is highlighted (green). Hydrogen atoms (except involved in hydrogen bonds) are omitted for clarity.

Despite the presence of three benzyl moieties in the mol­ecular structure of **1**, only weak π–π inter­actions are present in the crystal packing, with rather large centroid–centroid distances ranging from 4.4279 (11) to 5.9248 (9) Å. However, clear inter­molecular hydrogen bonds are found between the secondary amine N1—H1 hydrogen atoms and the thio­urea S1 atoms [N1—H1⋯S1 = 2.47 (3) Å; Table 1 ▸], linking the *N*,*N*,*N*′-tri­benzyl­thio­urea mol­ecules into infinite chains along the [001] direction, and forming columnar arrangements through alternating orientations of the mol­ecules (Fig. 3 ▸). Non-classical intra­molecular hydrogen bonds can be noticed between methyl C—H atoms of two benzyl groups and S1 atoms [C2—H2*A*⋯S1 = 2.70 Å; C9—H9*A*⋯S1 = 2.60 Å], as well as between benzene ring C—H atoms and tertiary amine N2 atoms [C15—H15⋯N2 = 2.51 Å; C22—H22⋯N2 = 2.58 Å]. Furthermore, several C—H⋯π contacts are observed in the range of 3.5419 (17)–3.8507 (19) Å, complementing the crystal packing.

**Figure 4:**
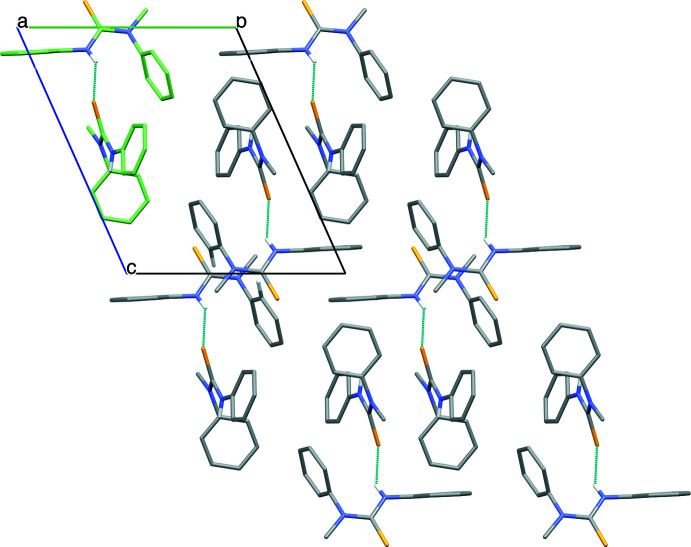
Packing in the structure of **2**, viewed down the *a* axis, showing the assembly of hydrogen-bonded pairs of mol­ecules, with one pair highlighted (green). Hydrogen atoms (except involved in hydrogen bonds) are omitted for clarity.

Analogous to **1**, the presence of two phenyl substituents in the mol­ecular structure of **2**, only leads to weak π–π inter­actions present in the crystal packing, with rather large centroid–centroid distances ranging from 4.8431 (13) to 5.9503 (12) Å. However, in this case, inter­molecular hydrogen bonds are formed between the two distinct mol­ecules in the asymmetric unit, *i.e.* between the secondary amine N1—H1 hydrogen atom of the first mol­ecule and the thio­urea S21 atom of the second mol­ecule [N1—H1⋯S21 = 2.58 (3) Å; Table 2 ▸], assembling the *N*-methyl-*N*,*N*′-di­phenyl­thio­urea mol­ecules into hydrogen-bonded pairs (Fig. 4 ▸). Non-classical intra­molecular hydrogen bonds can be noticed between the two methyl group C—H atoms, as well as phenyl ring C—H atoms, and S1/S21 atoms [C8—H8*B*⋯S1 = 2.65 Å; C28—H28*B*⋯S21 = 2.58 Å; C27—H27⋯S21 = 2.67 Å]. Additionally, an intra­molecular C=S⋯π contact is observed [C21=S21⋯*Cg*2 = 3.7115 (11) Å; *Cg*2 is the centroid of the C9–C14 ring]. Furthermore, several C—H⋯π contacts are observed in the range of 3.518 (2)–3.800 (2) Å, complementing the crystal packing.

**Figure 5:**
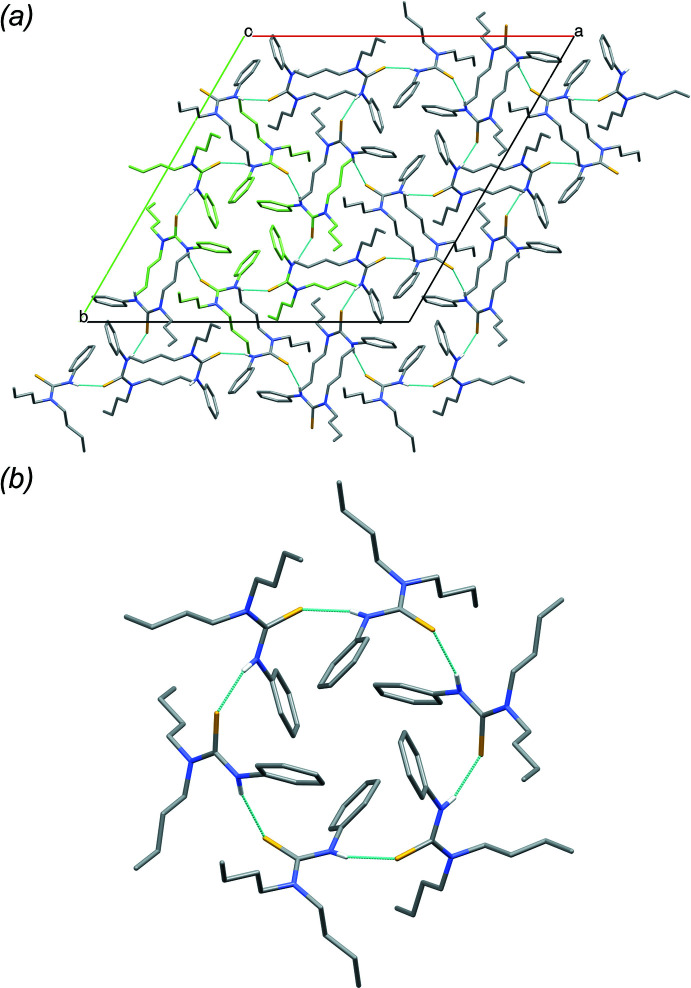
Packing in the structure of **3**, (*a*) viewed down the *c* axis, showing the hexa­mer ring assembly of mol­ecules, around the threefold rotoinversion axes, with one hexa­mer highlighted (green). (*b*) Detail of one hydrogen-bonded hexa­mer ring assembly. Hydrogen atoms (except involved in hydrogen bonds) are omitted for clarity.

Analogous to **1** and **2**, for **3**, only one type of weak π–π inter­action is present in the crystal packing, *i.e.* between symmetry-equivalent phenyl substituents, with a centroid–centroid distance of 4.9098 (10) Å. Inter­molecular hydrogen bonds are formed between the secondary amine N1—H1 hydrogen atoms and the thio­urea S1 atoms [N1—H1⋯S1 = 3.4656 (11) Å; Table 3 ▸], leading to a hexa­mer ring assembly of mol­ecules, around the threefold rotoinversion axes (Fig. 5 ▸). Non-classical intra- and inter­molecular hydrogen bonds can be noticed between two butyl CH_2_ groups and S1 [C8—H8*B*⋯S1 = 2.58 Å; C12—H12*A*⋯S1^i^; symmetry code: (i) − 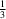 + *y*, 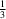 − *x* + *y*, 4/3 − *z*]. Only one C—H⋯π contact is observed [C4—H4⋯*Cg*1 = 3.6996 (17) Å; *Cg*1 is the centroid of the C2–C7 ring].

## Database survey

A survey of compounds, closely related to **1**, **2** and **3**, deposited with the Cambridge Structural Database (CSD 2021.1, version 5.42, updates of September 2021; Groom *et al.*, 2016 ▸) resulted in ten other thio­urea compounds, containing (substituted) benz­yl/phenyl rings on the secondary amine and (substituted) benz­yl/phenyl rings or alkyl groups on the tertiary amine, with refcodes HIFTIZ, HIFTOF, KUFQOS, KUFQOS01, KUFQOS02, POFJUR, QEMZOA, RAPNAA, RAQRAF and OYOSIH.

The structures with refcodes HIFTIX and HIFTOF are two unsymmetrical thio­urea derivatives, 1,1-dimethyl-3-*o*-tolyl­thio­urea and 1,1-diethyl-3-*o*-tolyl­thio­urea (Ramnathan *et al.*, 1996 ▸), containing *o*-tolyl groups as secondary amine substituents, while KUFQOS (Zhao *et al.*, 2008 ▸), KUFQOS01 (Panda *et al.*, 2017 ▸) and KUFQOS02 (Bhide *et al.*, 2021 ▸) represent the same structure of 1,1-dimethyl-3-phenyl­thio­urea. Halogen-substituted phenyl rings as secondary amine substituents are found for refcodes POFJUR and QEMZOA, which represent isomorphic structures of 3-(2-bromo-4-chloro­phen­yl)-1,1-di­methyl­thio­urea (El-Hiti *et al.*, 2014 ▸) and *N*′-(2-bromo-4-methyl­phen­yl)-*N*,*N*-di­methyl­thio­urea (El-Hiti *et al.*, 2018 ▸), respectively, while RAPNAA and RAQRAF represent structures of 3-(2-bromo­phen­yl)-1,1-di­methyl­thio­urea (El-Hiti *et al.*, 2017*a* ▸) and 3-(4-chloro­phen­yl)-1,1-di­methyl­thio­urea (El-Hiti *et al.*, 2017*b* ▸), respectively.

In all the above-mentioned structures, N—H⋯S hydrogen bonds link the mol­ecules into infinite chains, similar to **1**. This makes the reported structures of **2** and **3** unique in the sense that they show assemblies of hydrogen-bonded pairs and hexa­mer rings of mol­ecules, respectively.

As previously mentioned, OYOSIH (Rahman *et al.*, 2021 ▸) represents the same structure as **3**, although determined at room temperature and showed disorder of both butyl subs­tit­uents, as well as the presence of unknown solvent, which was treated by the SQUEEZE procedure in *PLATON* (Spek, 2015 ▸)

## Synthesis and crystallization

**General considerations** All manipulations were performed in air. All chemicals were used as received. Phenyl iso­thio­cyanate (97.0%) was purchased from Alfa Aesar. Chloro­form-*d*_1_ (stabilized with Ag, 99.8%D) was purchased from Carl Roth. Toluene (99.0%), aceto­nitrile (99.9%), *n*-hexane (99.0%), and abs. ethanol (99.8%) were purchased from Chem-Lab. Benzyl iso­thio­cyanate (98.0%), di­benzyl­amine (97.0%), *N*-methyl­aniline (98.0%), and di-*n*-butyl­amine (99.5%) were purchased from Sigma-Aldrich. Di­chloro­methane-*d*_2_ (99.8%D) was purchased from VWR. The thio­ureas were synthesized according to the procedure by Hendricks and Co-workers on a 30 mmol scale with the addition of a recrystallization step to purify the thio­urea (Hendricks *et al.*, 2015 ▸; Hamachi *et al.*, 2017 ▸).

**Synthesis of** ***N***, ***N***, ***N***’**-tri­benzyl­thio­urea (1):** A 40 mL vial was loaded with benzyl iso­thio­cyanate (4476.6 mg, 3.800 mL, 30 mmol, 1.0 eq.) in toluene (5 mL). To this, a solution of di­benzyl­amine (5918.4 mg, 5.800 mL, 30 mmol, 1.0 eq.) in toluene (5 mL) was added dropwise. The mixture was left to stir for 1 h at room temperature. Afterwards, the solvent was removed under reduced pressure, and the residual solid was recrystallized from hot aceto­nitrile which was cooled slowly (> 2 h) to room temperature and then to refrigerator temperature (275–281 K; > 2 h). The formed crystals were filtered off and extensively dried under dynamic vacuum to obtain white needle-like crystals (7.8 g, 75%), suitable for single-crystal X-ray diffraction analysis. **^1^H NMR** (400 MHz, CD_2_Cl_2_): δ 7.45–7.15 (*m*, 13H), δ 7.10–6.95 (*m*, 2H), δ 5.80 (*t*, *J* = 4.5 Hz, 1H), δ 5.00 (*s*, 4H), 4.80 (*d*, *J* = 2.6 Hz, 2H). **^13^C NMR** (100 MHz, CDCl_3_): δ 183.16, 137.78, 136.06, 129.17, 128.75, 128.03, 127.62, 127.19, 54.37, 50.79. **LC–MS** (API–ES) calculated for C_22_H_23_N_2_S [*M*+H]^+^ 347.16, found 347.1.

**Synthesis of** ***N*** **-methyl-** ***N***, ***N***’**-di­phenyl­thio­urea (2):** A 40 mL vial was loaded with phenyl iso­thio­cyanate (4055.7 mg, 3.585 mL, 30 mmol, 1.0 eq.) in toluene (5 mL). To this, a solution of *N*-methyl­aniline (3214.5 mg, 3.250 mL, 30 mmol, 1.0 eq.) in toluene (5 mL) was added dropwise. The mixture was left to stir for 6 h at 323 K, since the reaction with aniline derivatives elapses more sluggishly. Afterwards, the solvent was removed under reduced pressure, and the residual solid was recrystallized from a hot hexa­ne:ethanol (10:1) mixture which was cooled slowly (> 2 h) to room temperature and then to refrigerator temperature (275-281 K; > 2 h). The formed crystals were filtered off and extensively dried under dynamic vacuum to obtain white needle-like crystals (5.5 g, 76%), suitable for single-crystal X-ray diffraction analysis. **^1^H NMR** (400 MHz, CD_2_Cl_2_): δ 7.55–7.50 (*m*, 2H), δ 7.45–7.35 (*m*, 3H), δ 7.32–7.27 (*m*, 4H), δ 7.20–7.12 (*m*, 1H), δ 7.00 (*s*, 1H), δ 3.70 (*s*, 3H). **^13^C NMR** (100 MHz, CDCl_3_): δ 181.92, 143.52, 140.04, 131.05, 129.03, 128.76, 127.44, 126.21, 126.12, 43.73. **LC–MS** (API–ES) calculated for C_14_H_15_N_2_S [*M*+H]^+^ 243.10, found 243.1.

**Synthesis of *N*,*N*-di-*n*-butyl-*N*′-phenylthio­urea (3):** A 40 mL vial was loaded with phenyl iso­thio­cyanate (4055.7 mg, 3.585 mL, 30 mmol, 1.0 eq.) in toluene (5 mL). To this, a solution of di-*n*-butyl­amine (3877.2 mg, 5.055 mL, 30 mmol, 1.0 eq.) in toluene (5 mL) was added dropwise. The mixture was left to stir for 1 h at room temperature. Afterwards, the solvent was removed under reduced pressure, and the residual solid was recrystallized from a hot hexa­ne:ethanol (10:1) mixture which was cooled slowly (> 2 h) to room temperature and then to refrigerator temperature (275-281 K; > 2 h). The formed crystals were filtered off and extensively dried under dynamic vacuum to obtain white needle-like crystals (6.9 g, 87%), suitable for single-crystal X-ray diffraction analysis. **^1^H NMR** (400 MHz, CD_2_Cl_2_): δ 7.40–7.28 (*m*, 4H), δ 7.23–7.15 (*m*, 1H), δ 7.00 (*s*, 1H), δ 3.67 (*t*, *J* = 7.9 Hz, 4H), δ 1.71 (*quin*, *J* = 7.7 Hz, 4H), δ 1.38 (*six*, *J* = 7.9 Hz, 4H), δ 0.97 (*t*, *J* = 7.5 Hz, 6H). **^13^C NMR** (100 MHz, CDCl_3_): δ 181.56, 140.65, 128.85, 126.21, 125.84, 51.85, 29.95, 20.69, 14.06. **LC–MS** (API–ES) calc for C_15_H_25_N_2_S [*M*+H]^+^ 265.17, found 265.2.

**NMR spectroscopy.** Nuclear Magnetic Resonance (NMR) spectra of the synthesized organics were recorded on a Bruker 400 MHz. Chemical shifts (δ) are given in ppm and the residual solvent peak was used as an inter­nal standard (CDCl_3_: δH = 7.24 ppm, δC = 77.06 ppm, CD_2_Cl_2_: δH = 5.32 ppm, δC = 53.84 ppm). The signal multiplicity is denoted as follows: *s* (singlet), *d* (doublet), *t* (triplet), *quad* (quadruplet), *quin* (quintet), *six* (sextet), *m* (multiplet). Coupling constants are reported in Hertz (Hz). All resonances were corrected prior to integration by subtracting a background from the measured intensity. ^1^H, and ^13^C spectra were acquired using the standard pulse sequences from the Bruker library; zg30, and jmod (Attached Proton Test = APT), respectively.

**Mass spectroscopy.** Mass spectra (MS) were measured with an Agilent ESI single quadrupole detector type VL and an Agilent APCI single quadrupole detector type VL.

## Refinement

Crystal data, data collection and structure refinement details are summarized in Table 4 ▸. For all structures, the amine N–H hydrogen atoms could be located from a difference-Fourier electron-density map, and were further refined with isotropic temperature factors fixed at 1.2 times *U*_eq_ of the parent atoms. All other hydrogen atoms were refined in the riding mode with isotropic temperature factors fixed at 1.2 times *U*_eq_ of the parent atoms (1.5 times for methyl groups).

## Supplementary Material

Crystal structure: contains datablock(s) 1, 2, 3, New_Global_Publ_Block. DOI: 10.1107/S2056989022000147/vm2258sup1.cif

Structure factors: contains datablock(s) 1. DOI: 10.1107/S2056989022000147/vm22581sup2.hkl

Structure factors: contains datablock(s) 2. DOI: 10.1107/S2056989022000147/vm22582sup3.hkl

Structure factors: contains datablock(s) 3. DOI: 10.1107/S2056989022000147/vm22583sup4.hkl

CCDC references: 2132677, 2132676, 2132675

Additional supporting information:  crystallographic information; 3D view; checkCIF report

## Figures and Tables

**Table 1:** Hydrogen-bond geometry (Å, °) for **1** [Chem scheme1]

*D*—H⋯*A*	*D*—H	H⋯*A*	*D*⋯*A*	*D*—H⋯*A*
N1—H1⋯S1^i^	0.86 (3)	2.47 (3)	3.2044 (13)	145 (3)

Symmetry code: (i) x, -y+{\script{1\over 2}}, z+{\script{1\over 2}}.

**Table 2:** Hydrogen-bond geometry (Å, °) for **2** [Chem scheme1]

*D*—H⋯*A*	*D*—H	H⋯*A*	*D*⋯*A*	*D*—H⋯*A*
N1—H1⋯S21	0.86 (3)	2.58 (3)	3.3360 (16)	148 (3)

**Table 3:** Hydrogen-bond geometry (Å, °) for **3** [Chem scheme1]

*D*—H⋯*A*	*D*—H	H⋯*A*	*D*⋯*A*	*D*—H⋯*A*
N1—H1⋯S1^i^	0.86 (2)	2.62 (2)	3.4656 (11)	167 (2)
C12—H12*A*⋯S1^i^	0.99	2.67	3.6588 (13)	174

Symmetry code: (i) y-{\script{1\over 3}}, -x+y+{\script{1\over 3}}, -z+{\script{4\over 3}}.

**Table 4:** Experimental details

	**1**	**2**	**3**
Crystal data			
Chemical formula	C_22_H_22_N_2_S	C_14_H_14_N_2_S	C_15_H_24_N_2_S
*M*_r_	346.48	242.33	264.42
Crystal system, space group	Monoclinic, *P*2_1_/*c*	Triclinic, *P*\overline{1}	Trigonal, *R*\overline{3}
Temperature (K)	100	100	100
*a*, *b*, *c* (Å)	11.2378 (4), 14.7792 (5), 11.3165 (5)	9.8379 (6), 10.8014 (6), 13.2328 (6)	25.5231 (3), 25.5231 (3), 12.6225 (2)
α, β, γ (°)	90, 102.042 (3), 90	65.913 (5), 87.752 (4), 84.059 (5)	90, 90, 120
*V* (Å^3^)	1838.15 (12)	1276.82 (13)	7121.0 (2)
*Z*	4	4	18
Radiation type	Cu *K*α	Cu *K*α	Cu *K*α
μ (mm^−1^)	1.59	2.06	1.69
Crystal size (mm)	0.24 × 0.19 × 0.06	0.26 × 0.17 × 0.13	0.42 × 0.26 × 0.18

Data collection			
Diffractometer	SuperNova, Dual, Cu at home/near, Atlas	SuperNova, Dual, Cu at home/near, Atlas	SuperNova, Dual, Cu at home/near, Atlas
Absorption correction	Gaussian (*CrysAlis PRO*; Rigaku OD, 2019 ▸)	Gaussian (*CrysAlis PRO*; Rigaku OD, 2019 ▸)	Gaussian (*CrysAlis PRO*; Rigaku OD, 2019 ▸)
*T*_min_, *T*_max_	0.750, 1.000	0.687, 1.000	0.479, 1.000
No. of measured, independent and observed [*I* > 2σ(*I*)] reflections	16515, 3589, 3256	12208, 4818, 4298	14921, 3151, 3004
*R*_int_	0.060	0.028	0.021
(sin θ/λ)_max_ (Å^−1^)	0.624	0.623	0.622

Refinement			
*R*[*F* ^2^ > 2σ(*F* ^2^)], *wR*(*F* ^2^), *S*	0.043, 0.123, 1.06	0.049, 0.142, 1.06	0.032, 0.087, 1.06
No. of reflections	3589	4818	3151
No. of parameters	229	315	168
H-atom treatment	H atoms treated by a mixture of independent and constrained refinement	H atoms treated by a mixture of independent and constrained refinement	H atoms treated by a mixture of independent and constrained refinement
Δρ_max_, Δρ_min_ (e Å^−3^)	0.30, −0.31	0.58, −0.25	0.25, −0.18

Computer programs: *CrysAlis PRO* (Rigaku OD, 2019 ▸), *SHELXT2018/2* (Sheldrick, 2015*a* ▸), *SHELXL2018/3* (Sheldrick, 2015*b* ▸), *OLEX2* (Dolomanov *et al.*, 2009 ▸) and *Mercury* (Macrae *et al.*, 2020 ▸).

## supplementary crystallographic information

### N,N,N'-Tribenzylthiourea (1) Crystal data

**Table:**

C_22_H_22_N_2_S	*F*(000) = 736
*M**_r_* = 346.48	*D*_x_ = 1.252 Mg m^−^^3^
Monoclinic, *P*2_1_/*c*	Cu *K*α radiation, λ = 1.54184 Å
*a* = 11.2378 (4) Å	Cell parameters from 8187 reflections
*b* = 14.7792 (5) Å	θ = 4.0–74.0°
*c* = 11.3165 (5) Å	µ = 1.59 mm^−^^1^
β = 102.042 (3)°	*T* = 100 K
*V* = 1838.15 (12) Å^3^	Plate, clear colourless
*Z* = 4	0.24 × 0.19 × 0.06 mm

### N,N,N'-Tribenzylthiourea (1) Data collection

**Table:**

SuperNova, Dual, Cu at home/near, Atlas diffractometer	3589 independent reflections
Radiation source: micro-focus sealed X-ray tube, SuperNova (Cu) X-ray Source	3256 reflections with *I* > 2σ(*I*)
Mirror monochromator	*R*_int_ = 0.060
Detector resolution: 10.4839 pixels mm^-1^	θ_max_ = 74.2°, θ_min_ = 4.0°
ω scans	*h* = −13→13
Absorption correction: gaussian (CrysAlisPro; Rigaku OD, 2019)	*k* = −18→18
*T*_min_ = 0.750, *T*_max_ = 1.000	*l* = −11→13
16515 measured reflections	

### N,N,N'-Tribenzylthiourea (1) Refinement

**Table:**

Refinement on *F*^2^	Primary atom site location: dual
Least-squares matrix: full	Hydrogen site location: mixed
*R*[*F*^2^ > 2σ(*F*^2^)] = 0.043	H atoms treated by a mixture of independent and constrained refinement
*wR*(*F*^2^) = 0.123	*w* = 1/[σ^2^(*F*_o_^2^) + (0.0753*P*)^2^ + 0.4416*P*] where *P* = (*F*_o_^2^ + 2*F*_c_^2^)/3
*S* = 1.06	(Δ/σ)_max_ < 0.001
3589 reflections	Δρ_max_ = 0.30 e Å^−^^3^
229 parameters	Δρ_min_ = −0.31 e Å^−^^3^
0 restraints	Absolute structure: -

### N,N,N'-Tribenzylthiourea (1) Special details

**Table:**

Geometry. All esds (except the esd in the dihedral angle between two l.s. planes) are estimated using the full covariance matrix. The cell esds are taken into account individually in the estimation of esds in distances, angles and torsion angles; correlations between esds in cell parameters are only used when they are defined by crystal symmetry. An approximate (isotropic) treatment of cell esds is used for estimating esds involving l.s. planes.

### N,N,N'-Tribenzylthiourea (1) Fractional atomic coordinates and isotropic or equivalent isotropic displacement parameters (Å^2^)

**Table:**

	*x*	*y*	*z*	*U*_iso_*/*U*_eq_	
S1	0.10482 (3)	0.29165 (3)	0.32245 (3)	0.02217 (14)	
N1	0.12474 (11)	0.23113 (8)	0.54565 (11)	0.0198 (3)	
N2	0.25536 (10)	0.34685 (8)	0.52442 (11)	0.0191 (3)	
C1	0.16633 (12)	0.28879 (9)	0.47205 (13)	0.0177 (3)	
C2	0.04085 (13)	0.15697 (10)	0.50233 (13)	0.0209 (3)	
H2A	−0.024231	0.179223	0.435803	0.025*	
H2B	0.002213	0.136546	0.568717	0.025*	
C3	0.10428 (12)	0.07750 (10)	0.45767 (14)	0.0215 (3)	
C4	0.20059 (15)	0.03591 (12)	0.53478 (18)	0.0358 (4)	
H4	0.226181	0.057328	0.615227	0.043*	
C5	0.25996 (16)	−0.03682 (13)	0.4954 (2)	0.0519 (6)	
H5	0.326738	−0.064616	0.548228	0.062*	
H1	0.154 (3)	0.2304 (18)	0.622 (3)	0.062*	
C6	0.22149 (18)	−0.06872 (13)	0.3786 (2)	0.0494 (6)	
H6	0.262381	−0.118215	0.351143	0.059*	
C7	0.1240 (2)	−0.02889 (12)	0.30214 (19)	0.0433 (5)	
H7	0.096928	−0.051638	0.222546	0.052*	
C8	0.06532 (16)	0.04457 (11)	0.34141 (15)	0.0299 (4)	
H8	−0.001553	0.072197	0.288476	0.036*	
C9	0.31744 (12)	0.40391 (10)	0.45137 (13)	0.0202 (3)	
H9A	0.313944	0.374034	0.372425	0.024*	
H9B	0.404170	0.408858	0.492068	0.024*	
C10	0.26494 (12)	0.49838 (10)	0.42917 (13)	0.0190 (3)	
C11	0.31752 (13)	0.55802 (10)	0.35960 (14)	0.0242 (3)	
H11	0.384844	0.538845	0.327235	0.029*	
C12	0.27248 (15)	0.64526 (11)	0.33705 (15)	0.0281 (4)	
H12	0.308310	0.685134	0.288571	0.034*	
C13	0.17537 (14)	0.67414 (11)	0.38522 (15)	0.0276 (4)	
H13	0.144508	0.733810	0.370034	0.033*	
C14	0.12353 (13)	0.61561 (10)	0.45559 (15)	0.0249 (3)	
H14	0.057692	0.635536	0.489717	0.030*	
C15	0.16720 (12)	0.52792 (10)	0.47662 (13)	0.0211 (3)	
H15	0.130022	0.487852	0.523739	0.025*	
C16	0.29405 (13)	0.35662 (10)	0.65564 (13)	0.0209 (3)	
H16A	0.222424	0.347522	0.692503	0.025*	
H16B	0.323109	0.419334	0.673830	0.025*	
C17	0.39376 (12)	0.29173 (10)	0.71478 (14)	0.0195 (3)	
C18	0.45690 (13)	0.31034 (11)	0.83213 (14)	0.0230 (3)	
H18	0.438430	0.363493	0.871962	0.028*	
C19	0.54635 (13)	0.25185 (11)	0.89097 (14)	0.0264 (3)	
H19	0.589405	0.265389	0.970497	0.032*	
C20	0.57320 (13)	0.17348 (11)	0.83395 (15)	0.0263 (3)	
H20	0.634493	0.133350	0.874170	0.032*	
C21	0.50997 (13)	0.15434 (11)	0.71817 (15)	0.0244 (3)	
H21	0.527282	0.100429	0.679235	0.029*	
C22	0.42111 (13)	0.21354 (10)	0.65824 (14)	0.0221 (3)	
H22	0.379057	0.200265	0.578260	0.027*	

### N,N,N'-Tribenzylthiourea (1) Atomic displacement parameters (Å^2^)

**Table:**

	*U*^11^	*U*^22^	*U*^33^	*U*^12^	*U*^13^	*U*^23^
S1	0.0224 (2)	0.0317 (2)	0.0112 (2)	0.00067 (12)	0.00060 (14)	0.00133 (12)
N1	0.0220 (6)	0.0250 (6)	0.0118 (6)	0.0008 (5)	0.0020 (5)	0.0003 (5)
N2	0.0202 (6)	0.0220 (6)	0.0136 (6)	0.0015 (4)	0.0006 (4)	0.0009 (4)
C1	0.0162 (6)	0.0215 (7)	0.0153 (7)	0.0052 (5)	0.0028 (5)	0.0009 (5)
C2	0.0195 (7)	0.0251 (7)	0.0181 (7)	−0.0015 (5)	0.0041 (5)	0.0017 (6)
C3	0.0189 (7)	0.0240 (7)	0.0222 (8)	−0.0037 (5)	0.0057 (5)	0.0002 (6)
C4	0.0274 (8)	0.0291 (8)	0.0444 (11)	0.0016 (6)	−0.0073 (7)	−0.0044 (7)
C5	0.0243 (8)	0.0301 (10)	0.0956 (18)	0.0028 (7)	−0.0003 (9)	−0.0106 (10)
C6	0.0427 (11)	0.0253 (9)	0.0929 (18)	−0.0065 (7)	0.0434 (11)	−0.0113 (10)
C7	0.0719 (14)	0.0292 (9)	0.0394 (11)	−0.0185 (9)	0.0362 (10)	−0.0078 (8)
C8	0.0434 (9)	0.0277 (8)	0.0196 (8)	−0.0080 (7)	0.0090 (6)	0.0008 (6)
C9	0.0175 (6)	0.0251 (7)	0.0184 (7)	0.0009 (5)	0.0044 (5)	0.0013 (6)
C10	0.0178 (6)	0.0236 (7)	0.0141 (7)	−0.0007 (5)	−0.0002 (5)	−0.0016 (5)
C11	0.0242 (7)	0.0289 (8)	0.0194 (8)	−0.0031 (6)	0.0048 (5)	−0.0010 (6)
C12	0.0333 (8)	0.0272 (8)	0.0226 (9)	−0.0079 (6)	0.0029 (6)	0.0032 (6)
C13	0.0277 (8)	0.0216 (7)	0.0286 (9)	−0.0007 (6)	−0.0054 (6)	0.0010 (6)
C14	0.0190 (7)	0.0253 (8)	0.0283 (8)	0.0023 (5)	0.0001 (6)	−0.0020 (6)
C15	0.0180 (6)	0.0245 (7)	0.0196 (7)	−0.0009 (5)	0.0012 (5)	−0.0006 (6)
C16	0.0215 (7)	0.0251 (7)	0.0142 (8)	0.0027 (5)	−0.0003 (5)	−0.0012 (5)
C17	0.0166 (7)	0.0261 (7)	0.0156 (7)	−0.0013 (5)	0.0027 (5)	0.0031 (5)
C18	0.0226 (7)	0.0285 (7)	0.0167 (8)	−0.0028 (6)	0.0010 (5)	−0.0004 (6)
C19	0.0218 (7)	0.0373 (9)	0.0173 (8)	−0.0031 (6)	−0.0020 (5)	0.0038 (6)
C20	0.0169 (6)	0.0340 (8)	0.0264 (8)	0.0020 (6)	0.0012 (6)	0.0118 (7)
C21	0.0200 (7)	0.0279 (8)	0.0261 (9)	0.0030 (5)	0.0064 (6)	0.0027 (6)
C22	0.0192 (7)	0.0296 (8)	0.0167 (7)	0.0010 (5)	0.0019 (5)	0.0007 (6)

### N,N,N'-Tribenzylthiourea (1) Geometric parameters (Å, º)

**Table:**

S1—C1	1.6905 (14)	C10—C15	1.390 (2)
N1—C1	1.3419 (19)	C11—H11	0.9500
N1—C2	1.4621 (18)	C11—C12	1.389 (2)
N1—H1	0.85 (3)	C12—H12	0.9500
N2—C1	1.3569 (18)	C12—C13	1.385 (3)
N2—C9	1.4566 (19)	C13—H13	0.9500
N2—C16	1.4648 (19)	C13—C14	1.384 (2)
C2—H2A	0.9900	C14—H14	0.9500
C2—H2B	0.9900	C14—C15	1.389 (2)
C2—C3	1.514 (2)	C15—H15	0.9500
C3—C4	1.384 (2)	C16—H16A	0.9900
C3—C8	1.385 (2)	C16—H16B	0.9900
C4—H4	0.9500	C16—C17	1.5199 (19)
C4—C5	1.387 (3)	C17—C18	1.396 (2)
C5—H5	0.9500	C17—C22	1.386 (2)
C5—C6	1.384 (3)	C18—H18	0.9500
C6—H6	0.9500	C18—C19	1.386 (2)
C6—C7	1.378 (3)	C19—H19	0.9500
C7—H7	0.9500	C19—C20	1.389 (2)
C7—C8	1.391 (3)	C20—H20	0.9500
C8—H8	0.9500	C20—C21	1.383 (2)
C9—H9A	0.9900	C21—H21	0.9500
C9—H9B	0.9900	C21—C22	1.393 (2)
C9—C10	1.5162 (19)	C22—H22	0.9500
C10—C11	1.393 (2)		
			
C1—N1—C2	123.48 (12)	C15—C10—C11	118.84 (14)
C1—N1—H1	121.3 (18)	C10—C11—H11	119.7
C2—N1—H1	114.6 (18)	C12—C11—C10	120.66 (15)
C1—N2—C9	121.00 (12)	C12—C11—H11	119.7
C1—N2—C16	122.75 (13)	C11—C12—H12	120.0
C9—N2—C16	116.25 (11)	C13—C12—C11	120.03 (15)
N1—C1—S1	120.99 (11)	C13—C12—H12	120.0
N1—C1—N2	116.81 (13)	C12—C13—H13	120.2
N2—C1—S1	122.13 (11)	C14—C13—C12	119.64 (14)
N1—C2—H2A	109.2	C14—C13—H13	120.2
N1—C2—H2B	109.2	C13—C14—H14	119.8
N1—C2—C3	112.20 (11)	C13—C14—C15	120.39 (15)
H2A—C2—H2B	107.9	C15—C14—H14	119.8
C3—C2—H2A	109.2	C10—C15—H15	119.8
C3—C2—H2B	109.2	C14—C15—C10	120.43 (14)
C4—C3—C2	119.63 (14)	C14—C15—H15	119.8
C4—C3—C8	119.56 (15)	N2—C16—H16A	108.6
C8—C3—C2	120.79 (14)	N2—C16—H16B	108.6
C3—C4—H4	119.8	N2—C16—C17	114.82 (12)
C3—C4—C5	120.44 (18)	H16A—C16—H16B	107.5
C5—C4—H4	119.8	C17—C16—H16A	108.6
C4—C5—H5	120.1	C17—C16—H16B	108.6
C6—C5—C4	119.70 (19)	C18—C17—C16	118.44 (13)
C6—C5—H5	120.1	C22—C17—C16	122.47 (13)
C5—C6—H6	119.9	C22—C17—C18	119.05 (14)
C7—C6—C5	120.19 (17)	C17—C18—H18	119.7
C7—C6—H6	119.9	C19—C18—C17	120.51 (15)
C6—C7—H7	120.0	C19—C18—H18	119.7
C6—C7—C8	120.03 (18)	C18—C19—H19	119.9
C8—C7—H7	120.0	C18—C19—C20	120.19 (14)
C3—C8—C7	120.05 (17)	C20—C19—H19	119.9
C3—C8—H8	120.0	C19—C20—H20	120.3
C7—C8—H8	120.0	C21—C20—C19	119.47 (14)
N2—C9—H9A	108.7	C21—C20—H20	120.3
N2—C9—H9B	108.7	C20—C21—H21	119.8
N2—C9—C10	114.29 (12)	C20—C21—C22	120.48 (15)
H9A—C9—H9B	107.6	C22—C21—H21	119.8
C10—C9—H9A	108.7	C17—C22—C21	120.29 (14)
C10—C9—H9B	108.7	C17—C22—H22	119.9
C11—C10—C9	118.69 (13)	C21—C22—H22	119.9
C15—C10—C9	122.46 (13)		
			
N1—C2—C3—C4	57.70 (19)	C9—N2—C16—C17	−92.33 (15)
N1—C2—C3—C8	−123.79 (15)	C9—C10—C11—C12	−179.93 (13)
N2—C9—C10—C11	−179.60 (12)	C9—C10—C15—C14	178.87 (13)
N2—C9—C10—C15	1.06 (19)	C10—C11—C12—C13	0.9 (2)
N2—C16—C17—C18	164.26 (13)	C11—C10—C15—C14	−0.5 (2)
N2—C16—C17—C22	−18.1 (2)	C11—C12—C13—C14	−0.1 (2)
C1—N1—C2—C3	77.11 (17)	C12—C13—C14—C15	−0.9 (2)
C1—N2—C9—C10	95.30 (15)	C13—C14—C15—C10	1.2 (2)
C1—N2—C16—C17	88.11 (17)	C15—C10—C11—C12	−0.6 (2)
C2—N1—C1—S1	11.18 (19)	C16—N2—C1—S1	168.53 (10)
C2—N1—C1—N2	−171.82 (12)	C16—N2—C1—N1	−8.43 (19)
C2—C3—C4—C5	−179.84 (17)	C16—N2—C9—C10	−84.26 (14)
C2—C3—C8—C7	−179.52 (15)	C16—C17—C18—C19	178.27 (14)
C3—C4—C5—C6	−0.9 (3)	C16—C17—C22—C21	−177.37 (14)
C4—C3—C8—C7	−1.0 (2)	C17—C18—C19—C20	−0.6 (2)
C4—C5—C6—C7	−0.5 (3)	C18—C17—C22—C21	0.3 (2)
C5—C6—C7—C8	1.1 (3)	C18—C19—C20—C21	0.0 (2)
C6—C7—C8—C3	−0.4 (3)	C19—C20—C21—C22	0.8 (2)
C8—C3—C4—C5	1.6 (3)	C20—C21—C22—C17	−1.0 (2)
C9—N2—C1—S1	−11.01 (18)	C22—C17—C18—C19	0.5 (2)
C9—N2—C1—N1	172.03 (12)		

### N,N,N'-Tribenzylthiourea (1) Hydrogen-bond geometry (Å, º)

**Table:**

*D*—H···*A*	*D*—H	H···*A*	*D*···*A*	*D*—H···*A*
N1—H1···S1^i^	0.86 (3)	2.47 (3)	3.2044 (13)	145 (3)

Symmetry code: (i) *x*, −*y*+1/2, *z*+1/2.

### N-methyl-N,N'-Diphenylthiourea (2) Crystal data

**Table:**

C_14_H_14_N_2_S	*Z* = 4
*M**_r_* = 242.33	*F*(000) = 512
Triclinic, *P*1	*D*_x_ = 1.261 Mg m^−^^3^
*a* = 9.8379 (6) Å	Cu *K*α radiation, λ = 1.54184 Å
*b* = 10.8014 (6) Å	Cell parameters from 5889 reflections
*c* = 13.2328 (6) Å	θ = 3.6–73.7°
α = 65.913 (5)°	µ = 2.06 mm^−^^1^
β = 87.752 (4)°	*T* = 100 K
γ = 84.059 (5)°	Block, clear colourless
*V* = 1276.82 (13) Å^3^	0.26 × 0.17 × 0.13 mm

### N-methyl-N,N'-Diphenylthiourea (2) Data collection

**Table:**

SuperNova, Dual, Cu at home/near, Atlas diffractometer	4818 independent reflections
Radiation source: micro-focus sealed X-ray tube, SuperNova (Cu) X-ray Source	4298 reflections with *I* > 2σ(*I*)
Mirror monochromator	*R*_int_ = 0.028
Detector resolution: 10.4839 pixels mm^-1^	θ_max_ = 73.9°, θ_min_ = 3.7°
ω scans	*h* = −11→11
Absorption correction: gaussian (CrysAlisPro; Rigaku OD, 2019)	*k* = −13→12
*T*_min_ = 0.687, *T*_max_ = 1.000	*l* = −16→16
12208 measured reflections	

### N-methyl-N,N'-Diphenylthiourea (2) Refinement

**Table:**

Refinement on *F*^2^	Primary atom site location: dual
Least-squares matrix: full	Hydrogen site location: mixed
*R*[*F*^2^ > 2σ(*F*^2^)] = 0.049	H atoms treated by a mixture of independent and constrained refinement
*wR*(*F*^2^) = 0.142	*w* = 1/[σ^2^(*F*_o_^2^) + (0.0963*P*)^2^ + 0.1879*P*] where *P* = (*F*_o_^2^ + 2*F*_c_^2^)/3
*S* = 1.06	(Δ/σ)_max_ = 0.001
4818 reflections	Δρ_max_ = 0.58 e Å^−^^3^
315 parameters	Δρ_min_ = −0.25 e Å^−^^3^
0 restraints	

### N-methyl-N,N'-Diphenylthiourea (2) Special details

**Table:**

Geometry. All esds (except the esd in the dihedral angle between two l.s. planes) are estimated using the full covariance matrix. The cell esds are taken into account individually in the estimation of esds in distances, angles and torsion angles; correlations between esds in cell parameters are only used when they are defined by crystal symmetry. An approximate (isotropic) treatment of cell esds is used for estimating esds involving l.s. planes.

### N-methyl-N,N'-Diphenylthiourea (2) Fractional atomic coordinates and isotropic or equivalent isotropic displacement parameters (Å^2^)

**Table:**

	*x*	*y*	*z*	*U*_iso_*/*U*_eq_	
S1	0.87530 (5)	0.36219 (4)	−0.10166 (3)	0.03871 (15)	
N1	0.74118 (16)	0.27410 (15)	0.09073 (12)	0.0352 (3)	
N2	0.78346 (16)	0.49902 (15)	0.01836 (12)	0.0385 (3)	
C1	0.79648 (17)	0.37896 (17)	0.00793 (13)	0.0344 (4)	
C2	0.74183 (18)	0.13995 (17)	0.09466 (12)	0.0339 (4)	
C3	0.61818 (19)	0.08717 (18)	0.09855 (13)	0.0370 (4)	
H3	0.534808	0.141270	0.096050	0.044*	
C4	0.6160 (2)	−0.04473 (19)	0.10612 (14)	0.0415 (4)	
H4	0.531343	−0.081517	0.110231	0.050*	
C5	0.7377 (2)	−0.12244 (19)	0.10765 (14)	0.0431 (4)	
H5	0.736363	−0.211941	0.111191	0.052*	
C6	0.8613 (2)	−0.06994 (19)	0.10404 (14)	0.0419 (4)	
H6	0.944550	−0.123412	0.104909	0.050*	
C7	0.86365 (19)	0.06084 (18)	0.09914 (13)	0.0383 (4)	
H7	0.948411	0.095870	0.098860	0.046*	
C8	0.8263 (2)	0.62333 (19)	−0.06957 (16)	0.0466 (4)	
H8A	0.755527	0.661994	−0.127069	0.070*	
H8B	0.912030	0.601735	−0.101872	0.070*	
H8C	0.840149	0.689582	−0.038711	0.070*	
C9	0.71401 (18)	0.51678 (17)	0.11024 (14)	0.0357 (4)	
C10	0.78585 (18)	0.49184 (18)	0.20553 (15)	0.0379 (4)	
H10	0.879437	0.457338	0.212657	0.045*	
C11	0.7198 (2)	0.51775 (19)	0.29091 (15)	0.0429 (4)	
H11	0.768623	0.501230	0.356392	0.052*	
C12	0.5839 (2)	0.5672 (2)	0.28090 (17)	0.0461 (4)	
H12	0.539256	0.584186	0.339560	0.055*	
C13	0.5120 (2)	0.5923 (2)	0.18474 (19)	0.0470 (4)	
H13	0.418349	0.626521	0.177738	0.056*	
C14	0.5773 (2)	0.56728 (19)	0.09959 (17)	0.0420 (4)	
H14	0.528599	0.584619	0.033840	0.050*	
S21	0.52714 (5)	0.19752 (5)	0.30490 (4)	0.04442 (16)	
N21	0.47106 (17)	0.1816 (2)	0.50962 (13)	0.0464 (4)	
N22	0.69539 (17)	0.14244 (18)	0.47379 (14)	0.0456 (4)	
C21	0.5669 (2)	0.17492 (19)	0.43396 (15)	0.0408 (4)	
C22	0.33316 (19)	0.23455 (19)	0.49771 (14)	0.0380 (4)	
C23	0.2535 (2)	0.1894 (2)	0.59315 (14)	0.0438 (4)	
H23	0.292688	0.122901	0.660265	0.053*	
C24	0.1187 (2)	0.2401 (2)	0.59142 (16)	0.0491 (5)	
H24	0.065365	0.208041	0.656896	0.059*	
C25	0.0610 (2)	0.3379 (2)	0.49387 (18)	0.0520 (5)	
H25	−0.031833	0.373337	0.492102	0.062*	
C26	0.1397 (2)	0.3832 (2)	0.39947 (17)	0.0503 (5)	
H26	0.099883	0.449752	0.332619	0.060*	
C27	0.2752 (2)	0.3338 (2)	0.39998 (15)	0.0431 (4)	
H27	0.328299	0.367067	0.334463	0.052*	
C28	0.8129 (2)	0.1216 (2)	0.4083 (2)	0.0526 (5)	
H28A	0.862359	0.203300	0.379216	0.079*	
H28B	0.780515	0.104245	0.346634	0.079*	
H28C	0.874188	0.043352	0.455457	0.079*	
C29	0.72661 (18)	0.1336 (2)	0.58213 (15)	0.0418 (4)	
C30	0.7548 (2)	0.0084 (2)	0.6690 (2)	0.0541 (5)	
H30	0.756557	−0.072996	0.657398	0.065*	
C31	0.7807 (3)	0.0024 (2)	0.7737 (2)	0.0621 (6)	
H31	0.798209	−0.083740	0.833900	0.074*	
H1	0.688 (3)	0.290 (3)	0.138 (3)	0.074*	
H21	0.494 (3)	0.135 (3)	0.580 (3)	0.074*	
C32	0.7813 (2)	0.1195 (2)	0.79115 (17)	0.0497 (5)	
H32	0.798612	0.114241	0.862995	0.060*	
C33	0.7567 (2)	0.2446 (2)	0.70368 (16)	0.0438 (4)	
H33	0.759655	0.325717	0.714838	0.053*	
C34	0.72752 (19)	0.2523 (2)	0.59942 (15)	0.0430 (4)	
H34	0.708181	0.338601	0.539811	0.052*	

### N-methyl-N,N'-Diphenylthiourea (2) Atomic displacement parameters (Å^2^)

**Table:**

	*U*^11^	*U*^22^	*U*^33^	*U*^12^	*U*^13^	*U*^23^
S1	0.0441 (3)	0.0393 (3)	0.0306 (2)	−0.00590 (17)	0.00684 (17)	−0.01224 (18)
N1	0.0400 (8)	0.0323 (7)	0.0335 (7)	−0.0053 (5)	0.0067 (6)	−0.0139 (6)
N2	0.0462 (8)	0.0326 (7)	0.0349 (7)	−0.0076 (6)	0.0072 (6)	−0.0116 (6)
C1	0.0316 (8)	0.0362 (9)	0.0331 (7)	−0.0041 (6)	0.0001 (6)	−0.0117 (6)
C2	0.0425 (9)	0.0322 (8)	0.0262 (7)	−0.0054 (6)	0.0031 (6)	−0.0109 (6)
C3	0.0413 (9)	0.0372 (9)	0.0303 (7)	−0.0037 (7)	−0.0002 (6)	−0.0115 (6)
C4	0.0521 (11)	0.0398 (9)	0.0326 (8)	−0.0118 (8)	−0.0010 (7)	−0.0128 (7)
C5	0.0642 (12)	0.0328 (9)	0.0318 (8)	−0.0049 (8)	0.0016 (8)	−0.0128 (7)
C6	0.0506 (11)	0.0384 (9)	0.0323 (8)	0.0028 (7)	0.0058 (7)	−0.0118 (7)
C7	0.0419 (9)	0.0381 (9)	0.0314 (8)	−0.0045 (7)	0.0036 (7)	−0.0108 (7)
C8	0.0623 (12)	0.0345 (9)	0.0402 (9)	−0.0127 (8)	0.0105 (8)	−0.0116 (7)
C9	0.0397 (9)	0.0299 (8)	0.0378 (8)	−0.0068 (6)	0.0046 (7)	−0.0138 (7)
C10	0.0365 (9)	0.0344 (9)	0.0385 (8)	−0.0038 (6)	0.0026 (7)	−0.0108 (7)
C11	0.0516 (11)	0.0408 (10)	0.0353 (8)	−0.0099 (8)	0.0040 (7)	−0.0133 (7)
C12	0.0507 (11)	0.0430 (10)	0.0485 (10)	−0.0098 (8)	0.0144 (8)	−0.0225 (8)
C13	0.0361 (10)	0.0461 (11)	0.0633 (12)	−0.0038 (7)	0.0051 (8)	−0.0273 (9)
C14	0.0402 (10)	0.0383 (9)	0.0501 (10)	−0.0039 (7)	−0.0037 (8)	−0.0203 (8)
S21	0.0442 (3)	0.0584 (3)	0.0376 (2)	−0.0158 (2)	0.00781 (19)	−0.0247 (2)
N21	0.0393 (9)	0.0629 (11)	0.0288 (7)	0.0000 (7)	0.0020 (6)	−0.0115 (7)
N22	0.0390 (9)	0.0546 (10)	0.0426 (8)	−0.0017 (7)	0.0057 (6)	−0.0203 (7)
C21	0.0415 (10)	0.0406 (9)	0.0395 (9)	−0.0068 (7)	0.0062 (7)	−0.0154 (7)
C22	0.0373 (9)	0.0443 (9)	0.0324 (8)	−0.0068 (7)	0.0013 (7)	−0.0150 (7)
C23	0.0438 (10)	0.0516 (11)	0.0301 (8)	−0.0021 (8)	0.0011 (7)	−0.0112 (7)
C24	0.0422 (10)	0.0597 (12)	0.0366 (9)	−0.0036 (8)	0.0076 (7)	−0.0116 (8)
C25	0.0364 (10)	0.0549 (12)	0.0515 (11)	−0.0008 (8)	0.0011 (8)	−0.0092 (9)
C26	0.0444 (11)	0.0491 (11)	0.0417 (9)	−0.0040 (8)	−0.0022 (8)	−0.0025 (8)
C27	0.0417 (10)	0.0465 (10)	0.0340 (8)	−0.0088 (7)	0.0034 (7)	−0.0084 (7)
C28	0.0433 (11)	0.0587 (12)	0.0612 (12)	−0.0032 (9)	0.0114 (9)	−0.0312 (10)
C29	0.0321 (9)	0.0482 (10)	0.0412 (9)	−0.0036 (7)	0.0010 (7)	−0.0143 (8)
C30	0.0559 (13)	0.0411 (11)	0.0601 (12)	−0.0092 (9)	−0.0146 (10)	−0.0131 (9)
C31	0.0719 (15)	0.0467 (12)	0.0534 (12)	−0.0157 (10)	−0.0225 (11)	−0.0017 (9)
C32	0.0472 (11)	0.0539 (12)	0.0407 (9)	−0.0128 (8)	−0.0050 (8)	−0.0094 (8)
C33	0.0395 (10)	0.0489 (11)	0.0407 (9)	−0.0052 (7)	0.0061 (7)	−0.0161 (8)
C34	0.0399 (9)	0.0457 (10)	0.0356 (8)	0.0006 (7)	0.0055 (7)	−0.0101 (7)

### N-methyl-N,N'-Diphenylthiourea (2) Geometric parameters (Å, º)

**Table:**

S1—C1	1.6835 (17)	S21—C21	1.6798 (19)
N1—C1	1.359 (2)	N21—C21	1.367 (2)
N1—C2	1.428 (2)	N21—C22	1.405 (2)
N1—H1	0.86 (4)	N21—H21	0.88 (3)
N2—C1	1.352 (2)	N22—C21	1.345 (3)
N2—C8	1.462 (2)	N22—C28	1.472 (2)
N2—C9	1.442 (2)	N22—C29	1.441 (3)
C2—C3	1.386 (3)	C22—C23	1.395 (3)
C2—C7	1.386 (2)	C22—C27	1.395 (3)
C3—H3	0.9500	C23—H23	0.9500
C3—C4	1.389 (3)	C23—C24	1.379 (3)
C4—H4	0.9500	C24—H24	0.9500
C4—C5	1.385 (3)	C24—C25	1.387 (3)
C5—H5	0.9500	C25—H25	0.9500
C5—C6	1.385 (3)	C25—C26	1.379 (3)
C6—H6	0.9500	C26—H26	0.9500
C6—C7	1.390 (3)	C26—C27	1.383 (3)
C7—H7	0.9500	C27—H27	0.9500
C8—H8A	0.9800	C28—H28A	0.9800
C8—H8B	0.9800	C28—H28B	0.9800
C8—H8C	0.9800	C28—H28C	0.9800
C9—C10	1.384 (3)	C29—C30	1.381 (3)
C9—C14	1.388 (3)	C29—C34	1.392 (3)
C10—H10	0.9500	C30—H30	0.9500
C10—C11	1.393 (3)	C30—C31	1.393 (4)
C11—H11	0.9500	C31—H31	0.9500
C11—C12	1.377 (3)	C31—C32	1.376 (4)
C12—H12	0.9500	C32—H32	0.9500
C12—C13	1.394 (3)	C32—C33	1.379 (3)
C13—H13	0.9500	C33—H33	0.9500
C13—C14	1.382 (3)	C33—C34	1.387 (3)
C14—H14	0.9500	C34—H34	0.9500
			
C1—N1—C2	124.62 (14)	C21—N21—C22	131.73 (16)
C1—N1—H1	120 (2)	C21—N21—H21	116 (2)
C2—N1—H1	114 (2)	C22—N21—H21	112 (2)
C1—N2—C8	121.52 (15)	C21—N22—C28	122.70 (17)
C1—N2—C9	122.74 (14)	C21—N22—C29	121.26 (16)
C9—N2—C8	115.42 (14)	C29—N22—C28	115.95 (17)
N1—C1—S1	122.60 (13)	N21—C21—S21	123.08 (15)
N2—C1—S1	121.65 (13)	N22—C21—S21	122.99 (14)
N2—C1—N1	115.75 (15)	N22—C21—N21	113.87 (17)
C3—C2—N1	118.94 (15)	C23—C22—N21	116.39 (16)
C3—C2—C7	120.07 (17)	C23—C22—C27	119.09 (17)
C7—C2—N1	120.94 (16)	C27—C22—N21	124.39 (17)
C2—C3—H3	120.0	C22—C23—H23	119.5
C2—C3—C4	120.09 (17)	C24—C23—C22	120.92 (17)
C4—C3—H3	120.0	C24—C23—H23	119.5
C3—C4—H4	120.1	C23—C24—H24	120.1
C5—C4—C3	119.83 (19)	C23—C24—C25	119.87 (18)
C5—C4—H4	120.1	C25—C24—H24	120.1
C4—C5—H5	119.9	C24—C25—H25	120.3
C4—C5—C6	120.12 (18)	C26—C25—C24	119.35 (19)
C6—C5—H5	119.9	C26—C25—H25	120.3
C5—C6—H6	119.9	C25—C26—H26	119.3
C5—C6—C7	120.13 (17)	C25—C26—C27	121.47 (18)
C7—C6—H6	119.9	C27—C26—H26	119.3
C2—C7—C6	119.73 (18)	C22—C27—H27	120.3
C2—C7—H7	120.1	C26—C27—C22	119.30 (17)
C6—C7—H7	120.1	C26—C27—H27	120.3
N2—C8—H8A	109.5	N22—C28—H28A	109.5
N2—C8—H8B	109.5	N22—C28—H28B	109.5
N2—C8—H8C	109.5	N22—C28—H28C	109.5
H8A—C8—H8B	109.5	H28A—C28—H28B	109.5
H8A—C8—H8C	109.5	H28A—C28—H28C	109.5
H8B—C8—H8C	109.5	H28B—C28—H28C	109.5
C10—C9—N2	119.96 (16)	C30—C29—N22	120.46 (19)
C10—C9—C14	120.40 (16)	C30—C29—C34	119.83 (19)
C14—C9—N2	119.52 (16)	C34—C29—N22	119.71 (17)
C9—C10—H10	120.3	C29—C30—H30	120.3
C9—C10—C11	119.43 (17)	C29—C30—C31	119.4 (2)
C11—C10—H10	120.3	C31—C30—H30	120.3
C10—C11—H11	119.8	C30—C31—H31	119.5
C12—C11—C10	120.35 (18)	C32—C31—C30	120.9 (2)
C12—C11—H11	119.8	C32—C31—H31	119.5
C11—C12—H12	120.0	C31—C32—H32	120.2
C11—C12—C13	120.04 (17)	C31—C32—C33	119.6 (2)
C13—C12—H12	120.0	C33—C32—H32	120.2
C12—C13—H13	120.1	C32—C33—H33	119.9
C14—C13—C12	119.84 (18)	C32—C33—C34	120.2 (2)
C14—C13—H13	120.1	C34—C33—H33	119.9
C9—C14—H14	120.0	C29—C34—H34	120.0
C13—C14—C9	119.94 (18)	C33—C34—C29	120.03 (18)
C13—C14—H14	120.0	C33—C34—H34	120.0
			
N1—C2—C3—C4	−177.86 (15)	N21—C22—C23—C24	176.9 (2)
N1—C2—C7—C6	179.34 (15)	N21—C22—C27—C26	−176.8 (2)
N2—C9—C10—C11	175.88 (16)	N22—C29—C30—C31	−178.1 (2)
N2—C9—C14—C13	−176.16 (17)	N22—C29—C34—C33	179.60 (17)
C1—N1—C2—C3	−120.49 (18)	C21—N21—C22—C23	161.2 (2)
C1—N1—C2—C7	62.2 (2)	C21—N21—C22—C27	−23.1 (4)
C1—N2—C9—C10	89.0 (2)	C21—N22—C29—C30	105.4 (2)
C1—N2—C9—C14	−95.1 (2)	C21—N22—C29—C34	−74.2 (3)
C2—N1—C1—S1	−0.6 (2)	C22—N21—C21—S21	−16.0 (3)
C2—N1—C1—N2	178.92 (16)	C22—N21—C21—N22	166.9 (2)
C2—C3—C4—C5	−1.2 (3)	C22—C23—C24—C25	−0.4 (3)
C3—C2—C7—C6	2.0 (2)	C23—C22—C27—C26	−1.2 (3)
C3—C4—C5—C6	1.4 (3)	C23—C24—C25—C26	0.1 (4)
C4—C5—C6—C7	0.2 (3)	C24—C25—C26—C27	−0.4 (4)
C5—C6—C7—C2	−1.9 (3)	C25—C26—C27—C22	0.9 (3)
C7—C2—C3—C4	−0.5 (2)	C27—C22—C23—C24	1.0 (3)
C8—N2—C1—S1	5.5 (2)	C28—N22—C21—S21	−1.0 (3)
C8—N2—C1—N1	−174.01 (17)	C28—N22—C21—N21	176.15 (19)
C8—N2—C9—C10	−97.4 (2)	C28—N22—C29—C30	−78.0 (3)
C8—N2—C9—C14	78.5 (2)	C28—N22—C29—C34	102.4 (2)
C9—N2—C1—S1	178.71 (13)	C29—N22—C21—S21	175.44 (15)
C9—N2—C1—N1	−0.8 (2)	C29—N22—C21—N21	−7.4 (3)
C9—C10—C11—C12	0.3 (3)	C29—C30—C31—C32	−1.4 (4)
C10—C9—C14—C13	−0.3 (3)	C30—C29—C34—C33	0.0 (3)
C10—C11—C12—C13	−0.4 (3)	C30—C31—C32—C33	−0.3 (4)
C11—C12—C13—C14	0.1 (3)	C31—C32—C33—C34	1.9 (3)
C12—C13—C14—C9	0.2 (3)	C32—C33—C34—C29	−1.7 (3)
C14—C9—C10—C11	0.0 (3)	C34—C29—C30—C31	1.5 (3)

### N-methyl-N,N'-Diphenylthiourea (2) Hydrogen-bond geometry (Å, º)

**Table:**

*D*—H···*A*	*D*—H	H···*A*	*D*···*A*	*D*—H···*A*
N1—H1···S21	0.86 (3)	2.58 (3)	3.3360 (16)	148 (3)

### N,N-Di-n-butyl-N'-phenylthiourea (3) Crystal data

**Table:**

C_15_H_24_N_2_S	*D*_x_ = 1.110 Mg m^−^^3^
*M**_r_* = 264.42	Cu *K*α radiation, λ = 1.54184 Å
Trigonal, *R*3	Cell parameters from 8527 reflections
*a* = 25.5231 (3) Å	θ = 3.4–73.3°
*c* = 12.6225 (2) Å	µ = 1.69 mm^−^^1^
*V* = 7121.0 (2) Å^3^	*T* = 100 K
*Z* = 18	Block, clear colourless
*F*(000) = 2592	0.42 × 0.26 × 0.18 mm

### N,N-Di-n-butyl-N'-phenylthiourea (3) Data collection

**Table:**

SuperNova, Dual, Cu at home/near, Atlas diffractometer	3151 independent reflections
Radiation source: micro-focus sealed X-ray tube, SuperNova (Cu) X-ray Source	3004 reflections with *I* > 2σ(*I*)
Mirror monochromator	*R*_int_ = 0.021
Detector resolution: 10.4839 pixels mm^-1^	θ_max_ = 73.7°, θ_min_ = 3.5°
ω scans	*h* = −31→31
Absorption correction: gaussian (CrysAlisPro; Rigaku OD, 2019)	*k* = −28→28
*T*_min_ = 0.479, *T*_max_ = 1.000	*l* = −15→15
14921 measured reflections	

### N,N-Di-n-butyl-N'-phenylthiourea (3) Refinement

**Table:**

Refinement on *F*^2^	Primary atom site location: dual
Least-squares matrix: full	Hydrogen site location: mixed
*R*[*F*^2^ > 2σ(*F*^2^)] = 0.032	H atoms treated by a mixture of independent and constrained refinement
*wR*(*F*^2^) = 0.087	*w* = 1/[σ^2^(*F*_o_^2^) + (0.0479*P*)^2^ + 4.6459*P*] where *P* = (*F*_o_^2^ + 2*F*_c_^2^)/3
*S* = 1.06	(Δ/σ)_max_ = 0.001
3151 reflections	Δρ_max_ = 0.25 e Å^−^^3^
168 parameters	Δρ_min_ = −0.18 e Å^−^^3^
0 restraints	

### N,N-Di-n-butyl-N'-phenylthiourea (3) Special details

**Table:**

Geometry. All esds (except the esd in the dihedral angle between two l.s. planes) are estimated using the full covariance matrix. The cell esds are taken into account individually in the estimation of esds in distances, angles and torsion angles; correlations between esds in cell parameters are only used when they are defined by crystal symmetry. An approximate (isotropic) treatment of cell esds is used for estimating esds involving l.s. planes.

### N,N-Di-n-butyl-N'-phenylthiourea (3) Fractional atomic coordinates and isotropic or equivalent isotropic displacement parameters (Å^2^)

**Table:**

	*x*	*y*	*z*	*U*_iso_*/*U*_eq_	
S1	0.52061 (2)	0.89087 (2)	0.56407 (2)	0.03089 (10)	
N1	0.54435 (4)	0.80126 (4)	0.59489 (8)	0.0307 (2)	
N2	0.60326 (4)	0.88769 (4)	0.69020 (7)	0.0277 (2)	
C1	0.55829 (5)	0.85861 (5)	0.61987 (8)	0.0269 (2)	
C2	0.50126 (5)	0.76273 (5)	0.51835 (9)	0.0300 (2)	
C3	0.45844 (5)	0.70441 (6)	0.54908 (10)	0.0346 (3)	
H3	0.456357	0.692296	0.620843	0.041*	
C4	0.41868 (6)	0.66385 (6)	0.47470 (12)	0.0409 (3)	
H4	0.389778	0.623828	0.495424	0.049*	
C5	0.42119 (6)	0.68178 (6)	0.37040 (11)	0.0415 (3)	
H5	0.393836	0.654162	0.319656	0.050*	
C6	0.46357 (6)	0.73993 (7)	0.34018 (10)	0.0404 (3)	
H6	0.464950	0.752227	0.268704	0.049*	
C7	0.50412 (5)	0.78049 (6)	0.41355 (10)	0.0350 (3)	
H7	0.533631	0.820148	0.392150	0.042*	
C8	0.61573 (5)	0.94484 (5)	0.73935 (9)	0.0319 (2)	
H8A	0.602928	0.966811	0.690774	0.038*	
H8B	0.659778	0.970459	0.751515	0.038*	
C9	0.58233 (5)	0.93370 (5)	0.84445 (9)	0.0333 (3)	
H9A	0.538255	0.909261	0.831385	0.040*	
H9B	0.593836	0.910012	0.891379	0.040*	
C10	0.59590 (7)	0.99172 (6)	0.90032 (10)	0.0426 (3)	
H10A	0.582272	1.014382	0.855269	0.051*	
H10B	0.640170	1.017203	0.910118	0.051*	
C11	0.56494 (7)	0.97991 (7)	1.00770 (12)	0.0493 (3)	
H11A	0.520992	0.956005	0.998160	0.074*	
H11B	0.575502	1.018545	1.041429	0.074*	
H11C	0.578367	0.957572	1.052685	0.074*	
H1	0.5540 (9)	0.7814 (9)	0.6385 (15)	0.059*	
C12	0.64531 (5)	0.86653 (5)	0.71978 (9)	0.0288 (2)	
H12A	0.622637	0.821985	0.727847	0.035*	
H12B	0.664311	0.884432	0.788746	0.035*	
C13	0.69452 (5)	0.88425 (6)	0.63577 (9)	0.0342 (3)	
H13A	0.675206	0.869399	0.565789	0.041*	
H13B	0.719121	0.928910	0.632074	0.041*	
C14	0.73574 (6)	0.85867 (6)	0.65902 (10)	0.0359 (3)	
H14A	0.711309	0.813967	0.661602	0.043*	
H14B	0.754711	0.873062	0.729373	0.043*	
C15	0.78511 (6)	0.87735 (7)	0.57560 (12)	0.0477 (3)	
H15A	0.766520	0.863934	0.505610	0.071*	
H15B	0.809652	0.858661	0.591775	0.071*	
H15C	0.810909	0.921480	0.575771	0.071*	

### N,N-Di-n-butyl-N'-phenylthiourea (3) Atomic displacement parameters (Å^2^)

**Table:**

	*U*^11^	*U*^22^	*U*^33^	*U*^12^	*U*^13^	*U*^23^
S1	0.03175 (16)	0.03319 (16)	0.03491 (17)	0.02161 (12)	0.00062 (10)	0.00343 (10)
N1	0.0290 (5)	0.0276 (5)	0.0384 (5)	0.0163 (4)	−0.0062 (4)	−0.0022 (4)
N2	0.0267 (4)	0.0242 (4)	0.0329 (5)	0.0133 (4)	−0.0008 (4)	0.0005 (4)
C1	0.0244 (5)	0.0266 (5)	0.0307 (5)	0.0136 (4)	0.0036 (4)	0.0030 (4)
C2	0.0241 (5)	0.0301 (6)	0.0394 (6)	0.0164 (5)	−0.0024 (4)	−0.0056 (4)
C3	0.0294 (6)	0.0314 (6)	0.0453 (6)	0.0170 (5)	−0.0027 (5)	−0.0012 (5)
C4	0.0296 (6)	0.0315 (6)	0.0594 (8)	0.0137 (5)	−0.0045 (5)	−0.0067 (5)
C5	0.0308 (6)	0.0444 (7)	0.0491 (7)	0.0186 (6)	−0.0067 (5)	−0.0172 (6)
C6	0.0331 (6)	0.0525 (8)	0.0360 (6)	0.0217 (6)	0.0004 (5)	−0.0085 (5)
C7	0.0270 (5)	0.0380 (6)	0.0384 (6)	0.0150 (5)	0.0030 (4)	−0.0026 (5)
C8	0.0339 (6)	0.0232 (5)	0.0374 (6)	0.0134 (5)	−0.0031 (5)	−0.0009 (4)
C9	0.0316 (6)	0.0290 (6)	0.0391 (6)	0.0151 (5)	−0.0025 (5)	−0.0028 (5)
C10	0.0620 (9)	0.0358 (7)	0.0368 (6)	0.0294 (6)	−0.0076 (6)	−0.0041 (5)
C11	0.0566 (9)	0.0548 (8)	0.0453 (7)	0.0344 (7)	−0.0024 (6)	−0.0111 (6)
C12	0.0263 (5)	0.0261 (5)	0.0338 (5)	0.0130 (4)	−0.0032 (4)	0.0016 (4)
C13	0.0315 (6)	0.0361 (6)	0.0365 (6)	0.0180 (5)	0.0006 (5)	0.0029 (5)
C14	0.0347 (6)	0.0396 (6)	0.0372 (6)	0.0214 (5)	−0.0042 (5)	−0.0046 (5)
C15	0.0337 (7)	0.0558 (8)	0.0531 (8)	0.0220 (6)	0.0010 (6)	−0.0082 (6)

### N,N-Di-n-butyl-N'-phenylthiourea (3) Geometric parameters (Å, º)

**Table:**

S1—C1	1.7004 (11)	C9—H9A	0.9900
N1—C1	1.3594 (15)	C9—H9B	0.9900
N1—C2	1.4241 (15)	C9—C10	1.5157 (17)
N1—H1	0.86 (2)	C10—H10A	0.9900
N2—C1	1.3432 (15)	C10—H10B	0.9900
N2—C8	1.4663 (14)	C10—C11	1.521 (2)
N2—C12	1.4705 (14)	C11—H11A	0.9800
C2—C3	1.3907 (17)	C11—H11B	0.9800
C2—C7	1.3885 (17)	C11—H11C	0.9800
C3—H3	0.9500	C12—H12A	0.9900
C3—C4	1.3901 (18)	C12—H12B	0.9900
C4—H4	0.9500	C12—C13	1.5293 (16)
C4—C5	1.385 (2)	C13—H13A	0.9900
C5—H5	0.9500	C13—H13B	0.9900
C5—C6	1.383 (2)	C13—C14	1.5186 (17)
C6—H6	0.9500	C14—H14A	0.9900
C6—C7	1.3888 (18)	C14—H14B	0.9900
C7—H7	0.9500	C14—C15	1.5242 (18)
C8—H8A	0.9900	C15—H15A	0.9800
C8—H8B	0.9900	C15—H15B	0.9800
C8—C9	1.5249 (17)	C15—H15C	0.9800
			
C1—N1—C2	126.66 (10)	C10—C9—H9B	109.0
C1—N1—H1	119.1 (13)	C9—C10—H10A	109.2
C2—N1—H1	112.1 (13)	C9—C10—H10B	109.2
C1—N2—C8	121.99 (9)	C9—C10—C11	112.25 (12)
C1—N2—C12	122.95 (9)	H10A—C10—H10B	107.9
C8—N2—C12	115.00 (9)	C11—C10—H10A	109.2
N1—C1—S1	121.22 (8)	C11—C10—H10B	109.2
N2—C1—S1	122.70 (8)	C10—C11—H11A	109.5
N2—C1—N1	116.08 (10)	C10—C11—H11B	109.5
C3—C2—N1	118.18 (11)	C10—C11—H11C	109.5
C7—C2—N1	121.64 (11)	H11A—C11—H11B	109.5
C7—C2—C3	120.00 (11)	H11A—C11—H11C	109.5
C2—C3—H3	120.0	H11B—C11—H11C	109.5
C4—C3—C2	119.94 (12)	N2—C12—H12A	109.4
C4—C3—H3	120.0	N2—C12—H12B	109.4
C3—C4—H4	120.0	N2—C12—C13	110.96 (9)
C5—C4—C3	120.01 (12)	H12A—C12—H12B	108.0
C5—C4—H4	120.0	C13—C12—H12A	109.4
C4—C5—H5	120.0	C13—C12—H12B	109.4
C6—C5—C4	119.94 (12)	C12—C13—H13A	109.1
C6—C5—H5	120.0	C12—C13—H13B	109.1
C5—C6—H6	119.8	H13A—C13—H13B	107.8
C5—C6—C7	120.48 (13)	C14—C13—C12	112.46 (10)
C7—C6—H6	119.8	C14—C13—H13A	109.1
C2—C7—C6	119.61 (12)	C14—C13—H13B	109.1
C2—C7—H7	120.2	C13—C14—H14A	109.2
C6—C7—H7	120.2	C13—C14—H14B	109.2
N2—C8—H8A	109.4	C13—C14—C15	111.96 (11)
N2—C8—H8B	109.4	H14A—C14—H14B	107.9
N2—C8—C9	111.07 (9)	C15—C14—H14A	109.2
H8A—C8—H8B	108.0	C15—C14—H14B	109.2
C9—C8—H8A	109.4	C14—C15—H15A	109.5
C9—C8—H8B	109.4	C14—C15—H15B	109.5
C8—C9—H9A	109.0	C14—C15—H15C	109.5
C8—C9—H9B	109.0	H15A—C15—H15B	109.5
H9A—C9—H9B	107.8	H15A—C15—H15C	109.5
C10—C9—C8	112.91 (10)	H15B—C15—H15C	109.5
C10—C9—H9A	109.0		
			
N1—C2—C3—C4	−174.90 (10)	C3—C4—C5—C6	0.45 (19)
N1—C2—C7—C6	175.74 (11)	C4—C5—C6—C7	0.57 (19)
N2—C8—C9—C10	177.68 (10)	C5—C6—C7—C2	−1.16 (19)
N2—C12—C13—C14	−175.23 (10)	C7—C2—C3—C4	0.27 (17)
C1—N1—C2—C3	−129.37 (12)	C8—N2—C1—S1	11.78 (15)
C1—N1—C2—C7	55.54 (16)	C8—N2—C1—N1	−168.43 (10)
C1—N2—C8—C9	92.75 (12)	C8—N2—C12—C13	−97.50 (11)
C1—N2—C12—C13	79.64 (13)	C8—C9—C10—C11	−176.92 (11)
C2—N1—C1—S1	3.80 (16)	C12—N2—C1—S1	−165.16 (8)
C2—N1—C1—N2	−176.00 (10)	C12—N2—C1—N1	14.63 (15)
C2—C3—C4—C5	−0.87 (18)	C12—N2—C8—C9	−90.08 (11)
C3—C2—C7—C6	0.74 (17)	C12—C13—C14—C15	−179.20 (11)

### N,N-Di-n-butyl-N'-phenylthiourea (3) Hydrogen-bond geometry (Å, º)

**Table:**

*D*—H···*A*	*D*—H	H···*A*	*D*···*A*	*D*—H···*A*
N1—H1···S1^i^	0.86 (2)	2.62 (2)	3.4656 (11)	167 (2)
C12—H12*A*···S1^i^	0.99	2.67	3.6588 (13)	174

Symmetry code: (i) *y*−1/3, −*x*+*y*+1/3, −*z*+4/3.
